# Inflammasomes and Cancer: The Dynamic Role of the Inflammasome in Tumor Development

**DOI:** 10.3389/fimmu.2017.01132

**Published:** 2017-09-12

**Authors:** Melvin Kantono, Beichu Guo

**Affiliations:** ^1^Department of Microbiology and Immunology, Medical University of South Carolina (MUSC), Charleston, SC, United States; ^2^Hollings Cancer Center, Medical University of South Carolina (MUSC), Charleston, SC, United States

**Keywords:** inflammasome, tumor microenvironments, NOD-like receptors, IL-1, tumor, inflammation, immunotherapy

## Abstract

Chronic Inflammation in tumor microenvironments is not only associated with various stages of tumor development, but also has significant impacts on tumor immunity and immunotherapy. Inflammasome are an important innate immune pathway critical for the production of active IL-1β and interleukin 18, as well as the induction of pyroptosis. Although extensive studies have demonstrated that inflammasomes play a vital role in infectious and autoimmune diseases, their role in tumor progression remains elusive. Multiple studies using a colitis-associated colon cancer model show that inflammasome components provide protection against the development of colon cancer. However, very recent studies demonstrate that inflammasomes promote tumor progression in skin and breast cancer. These results indicate that inflammasomes can promote and suppress tumor development depending on types of tumors, specific inflammasomes involved, and downstream effector molecules. The complicated role of inflammasomes raises new opportunities and challenges to manipulate inflammasome pathways in the treatment of cancer.

## Introduction

**Figure 1:**
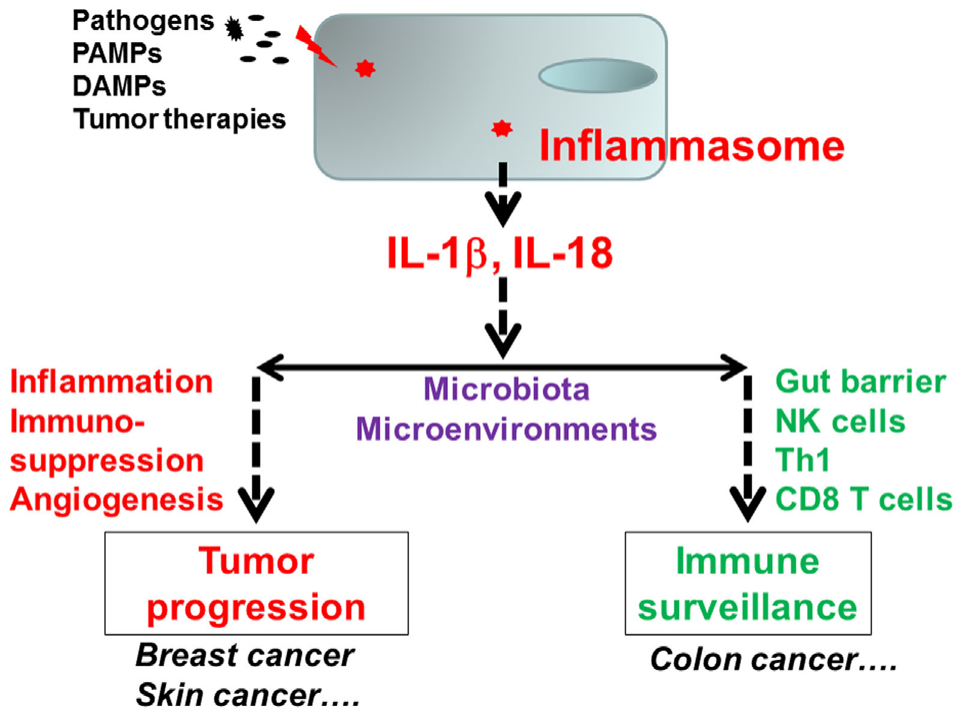
The dynamic role of the inflammasome in tumor development. The inflammasomes can promote or inhibit tumor progression depending on context. In colitis-associated colon cancer, inflammasome-derived interleukin 18 (IL-18) supports intestinal barrier function and induces tumor surveillance at the intestinal mucosal surface. For breast cancer and skin cancer, IL-1β-induced inflammation and immunosuppression promote tumor growth and metastasis. Tumor microenvironments and gut microbiota may also influence tumor progression and host antitumor immunity.

Emerging evidence indicates that chronic inflammation plays an important role at all stages of tumor development, including initiation, growth, invasion, and metastasis (1–7). As part of the immune surveillance system, various innate immune pathways may engage with cellular components released from dead tumor cells due to hypoxia, chemotherapy, radiotherapy, or an immune attack (8–11). The innate immune cells activated by tumors or tumor components may induce antitumor immunity through the recruitment of effector cells or promote tumor development by providing a pro-inflammatory environment (Figure 1). While there are numerous studies on the involvement of toll-like receptors (TLRs) or interferon (IFN) pathways in tumor development (9, 12–16), the role of inflammasomes in tumor development is poorly characterized. The inflammasome is a novel innate immune pathway involved in the production of active IL-1β and interleukin 18 (IL-18), which are potent inflammatory cytokines (11, 17–21). Extensive evidence indicates that inflammasomes play a vital role in pathogen infections and autoimmune diseases. However, their role in tumor progression remains unclear. Many published studies use colitis-induced colon cancer as an animal model to investigate the role of inflammasomes in cancer. Results from those studies indicate that inflammasome components provide protection against tumorigenesis in colitis-associated colon cancer (22–29). Yet recent studies from our group and others demonstrate that inflammasomes can promote tumor development in certain types of cancer (30–32).

In this review, we will discuss how the inflammasome and its effector pathways influence the pathogenesis of various types of cancer (Figure 1). The first part of the paper provides basic information about inflammasomes. Subsequently, we use a colitis-associated colon cancer model to illustrate the protect role of inflammasomes against tumors. Then, we utilize skin and breast cancer models to demonstrate the tumor-promoting effects of inflammasomes. Finally, we list some questions about inflammasomes and cancer in the perspective section.

### Inflammasomes

The inflammasome is a major component of the innate immune system that functions to induce maturation of inflammatory cytokines such as Interleukin 1 (IL-1β) and IL-18 in response to infection or autogenous danger signals (33–39). An inflammasome is a multimolecular complex, composed of a NOD-like protein (NLR), the adaptor apoptosis-associated speck-like protein containing a caspase recruitment domain (ASC), and caspase-1. NLRs belong to host pattern-recognition receptors (PRRs) that recognize pathogen-associated molecular patterns (PAMPs) from bacteria or viruses to initiate the innate immune response (11, 20, 40–42). These PRRs can be found on the membrane surface, e.g., TLRs and C-type lectin receptors, or intracellularly, e.g., NOD-like receptors (NLRs) and RIG-I-like receptors. Like TLRs, NLRPs also interact with endogenous ligands or damage-associated molecular patterns (DAMPs) from normal host tissues or tumor cells to induce autoimmune diseases or an antitumor response. In recent years, NLRP proteins have attracted lots of attention because some of them can form inflammasomes.

The NLRs generally comprise of a caspase recruitment domain (CARD), or pyrin (PYD) at the N-terminus, a nucleotide-binding and oligomerization domain (NBD or NACHT) in the middle, and leucine-rich repeat (LRR) at the C-terminus (23, 33, 43, 44). The CARD or PYD interacts with the PYD domain in ASC or other downstream signaling molecules, NACHT is involved in oligomerization and other regulatory functions, whereas LRR functions as a sensor or signal receiver from PAMPs and DAMPs. Production of mature or active IL-1β needs two signals: the first signal is initiated by TLR ligands or endogenous molecules to induce the expression of pro-IL-1β proteins; the second is triggered by very diverse stimuli activating inflammasomes, leading to caspase-1-dependent conversion of pro-IL-1β to mature IL-1β. Upon engagement of endogenous or exogenous stimuli, NLRP proteins interact with ASC and caspase-1 and undergo oligomerization, eventually forming a huge signaling complex. When pro-caspase-1 is associated with ASC and NLRPs, it undergoes self-cleavage to form an active form of caspase-1 enzyme, which in turn process pro-IL-1 and pro-IL-18 proteins into their active forms. Activation of inflammasomes also leads to a form of inflammatory cell death, termed as pyroptosis, through a pore-forming protein gasdermin D (45–48). NLRs have about 22 family members in the human genome and more than 30 members in the mouse genome. Many, but not all, NLRs can form inflammasomes. While the NLR, pyrin domain containing 3 (NLRP3) inflammasome is the most studied one in this group of signaling complexes, other inflammasomes, including NLRP1, NLRC4, and NLRP12 inflammasomes, have also been identified (33–39). In addition to NLRs, the HIN200 family members absent in myeloma 2 (AIM2) and IFN-inducible protein 16 (IFI16) can assemble inflammasomes with ASC and caspase-1 (49, 50). AIM2 has been shown to form an inflammasome in response to bacterial DNA, viral DNA, or endogenous DNA released during cellular damage (49–51). Similarly, IFI16 recruits ASC and caspase-1 to assemble an inflammasome upon sensing DNA from Kaposi sarcoma-associated herpes virus (52–55). Because of its potential to recognize host DNA, AIM2 may contribute to the development of autoimmune diseases, including systemic lupus erythematosus, psoriasis, and arthritis. Furthermore, recent studies show that AIM2 is required to mediate protection against colorectal cancer (56–61). Generally, each type of inflammasomes, except NLRP3, recognizes defined molecular patterns from pathogens. For example, NLRC4 senses cytoplasmic flagellin and bacterial type III secretion apparatus from Gram-negative bacteria such as *Salmonella typhimurium*. NLRP1 can sense muramyl dipeptide, and *Bacillus anthracis* lethal toxin. AIM2 recognizes cytoplasmic DNA from host or pathogens (33–39). Although many stimuli with very diverse and unrelated molecular structures can trigger the activation of the NLRP3 inflammasome, the underlying molecular mechanisms remain elusive.

NOD-like receptor proteins have been shown to be associated with various autoimmune or inflammatory diseases. For example, NOD1 and NOD2 have been implicated in the pathogenesis of inflammatory bowel disease (IBD) (62, 63). Mutations in the human NLRP3 gene is associated with a group of autoimmune diseases termed as cold-induced auto-inflammatory syndrome, including familial cold-induced auto-inflammatory syndrome, Muckle–Wells syndrome, and NOMID/CINCA (neonatal onset multisystem inflammatory disorder or chronic infantile neurologic cutaneous and articular syndrome) (34, 64–67). Accumulating evidence shows that NLRP3 inflammasome is involved in a wide array of autoimmune and inflammatory diseases, such as IBD, liver steatosis, cardiovascular disease, rheumatoid arthritis, type one diabetes, and neurologic diseases (21, 23, 38, 43, 64, 68–71).

IL-1β and IL-18 are potent inflammatory cytokines that trigger various signaling pathways, including NF-κB, MAPK, and PI3K pathways (72–76). They are members of the IL-1 family of cytokines produced by macrophages and other cells during an immune response. Previous clinical studies also reveal that increased IL-1β in tumor tissues is associated with poorer prognosis (72, 77–80). Although inflammasomes are essential for host defense against pathogens and contribute to autoimmune diseases, their roles in tumor progression remain controversial. Results from published studies have shown that inflammasomes can inhibit or promote tumor growth and progression. Currently, we have very limited knowledge of the mechanisms responsible for inflammasome activation during tumor development and therapies.

### Inflammasomes Inhibit Cancer Development in Colitis-Associated Colon Cancer

A series of studies using various NLRP or caspase-1-deficient mice have reported that inflammasome activities protect mice from colitis-associated colon cancer (CAC) induced by azoxymethane/dextran sodium sulfate (AOM/DSS) (22–29). In this model, DSS in drinking water causes damage to the epithelial barrier, resulting in massive inflammation induced by gut microflora. AOM is a potent carcinogen causing DNA damage in epithelial cells. Repeating DSS administration cause chronic inflammation, which promotes colorectal cancer development in cells harboring mutations elicited by AOM. Results from these studies show that mice deficient for inflammasome components, including NLRP3, caspase-1, NLRP1, NLRP6, and NLRC4, are highly susceptible to colitis-associated colon cancer induced by AOM/DSS by displaying severe intestinal inflammation, and increased number of colon polyps (22–29).

While inflammasome-deficient mice are generally susceptible to DSS-induced colitis, the results on a particular NLRP may vary. For instance, Hu et al. showed NLRC4 and caspase-1 control colitis-associated tumorigenesis (81). In the AOM/DSS-induced colorectal cancer model, NLRC4 and caspase-1 KO mice exhibited increased tumor load and tumor number per mice. However, the authors found no difference in colitis-associated colon cancer between the NLRP3-deficient and WT mice (81). In contrast, Allen et al. found that NLRC4 had no protective role in tumorigenesis, compared to WT mice. Instead, NLRP3 expression in hematopoietic cell compartment is essential for protection against colon cancer (25). This discrepancy may be due to experimental conditions or micro biota associated with mouse colonies.

Surprisingly, the results from these studies indicate that IL-18, but not IL-1β, plays a major role in suppressing colitis. Further mechanistic studies suggest that inflammasome-mediated IL-18 is critical for intestinal tissue repair and remodeling as discussed below. Moreover, injection of recombinant IL-18 could ameliorate the severity of DSS-induced colitis in inflammasome-deficient mice. These results suggest that during this chemically induced inflammation, IL-18 produced during inflammasome activation is vital for the homeostasis of the epithelial barrier in the intestinal tissues. This conclusion is further supported by studies showing that IL-18 KO or IL-18R KO mice are also highly susceptible to DSS-induced colitis and AOM/DSS-induced colon cancer (82, 83). All these studies highlight the importance of inflammasome-dependent IL-18 production in suppressing colorectal tumorigenesis.

### Role of IL-18 and Microbiota in Inflammation-Associated Colon Cancer

The interaction of microbiota and the intestinal system is essential for maintaining host homeostasis and development of mucosal immunity (84–87). The human body, especially the gastrointestinal tract, is colonized by trillions of bacteria. While the commensal microorganisms are essential for the homeostasis of intestinal system and the development of host immune system, altered community representation and function of microbial species in the gut ecosystem, a state called dysbiosis, could induce intestinal inflammation and epithelial neoplasia (85, 87–95).

The commensal microbiota and bacterial products are sensed and monitored by epithelial cells and innate immune cells *via* innate receptors, including TLRs and NLRs. As mentioned above, inflammasome-mediated IL-18 is critical for intestinal tissue remodeling and barrier function, which has significant impacts on intestinal inflammation, gut microbiota, and even the systemic immunity. At steady state, commensal bacteria and their products induce inflammasome activation and IL-18 production in the colon that supports intestinal barrier function and prevents commensal dysbiosis (11, 25, 82, 96–98). Deficiency in inflammasome components leads to reduced production of IL-18, resulting in impaired epithelial remodeling and regeneration. Loss of barrier function causes increased commensal bacteria penetration, and enhanced inflammation, which may promote tumorigenesis and tumor growth. Dysbiosis has been observed in mice deficient for inflammasome components, including NLRP6, ASC, caspase-1, and IL-18 (25, 82, 96–99). For example, it has been proposed that NLRP6 is required for the maintenance of both composition and distribution of commensal bacteria in the gut. NLRP6 KO mice show altered microbial composition, exacerbated colitis upon chemically induced damage to the epithelial barrier, and inceased incidence of inflammation-associated colon cancer (100–102). While solid evidence has demonstrated the protective roles of inflammasomes and IL-18 in AOM/DSS-induced colon cancer, it would be informative to determine whether inflammasomes, IL-1β or IL-18 inhibit or promote colon cancer development in chronic or genetic colon cancer models as well as in human colorectal cancer patients.

Although several studies show that inflammasome deficiency leads to aberrations in microbial ecology or dysbiosis, a recent report by Mamantopoulos et al. challenges the role of inflammasomes in gut microbiota and colitis (103). Mamantopoulos et al. tested whether inflammasomes shape gut ecology by carefully analyzing NLRP6-deficient mice and littermate controls. Their results show that NLRP6 inflammasome deficiency does not affect gut microbiota composition and DSS-induced colitis when controlling for non-genetic confounders (103). This finding raises questions about previous publications related to the role of inflammasomes in controlling intestinal ecology. Therefore, further studies are required to verify whether inflammasomes shape intestinal microbiota ecology, and how the dysbiotic microbiota associated with inflammasome deficiency affect tumorigenesis and tumor progression.

### Cross Talk of Inflammasome Mediators in Colitis and Colon Cancer

While numerous studies indicate the protective role of inflammasomes and IL-18 in DSS-induced colitis, recent studies demonstrate inflammasome-mediated IL-1β drives chronic inflammation in IL-10 KO mice and in human patients with IL-10R deficiency (38, 104, 105). It seems that IL-1β and IL-18 may have different roles in intestinal inflammation. As IL-1 and IL-18 are main inflammatory mediators processed by inflammasomes, it remains unclear how the host immune system integrates IL-1 and IL-18 signals during colitis and inflammation-associated colon cancer. Currently, there are a few studies on this topic. One possible mechanism is the differential transcriptional regulation of those two cytokines. Zhu and Kanneganti found that IL-18 expression was increased and sustained after TLR stimulation, whereas IL-1β expression was upregulated but not sustained upon stimulation *in vitro*. Furthermore, induction of IL-18 in macrophages requires type I IFN signaling (106). Interestingly, previous studies demonstrate that type I IFN suppresses transcription of IL-1β gene in macrophages (107). Additionally, IL-1β and IL-18 may influence intestinal inflammation through exerting distinct effects on T cell subsets. Accumulating evidence suggests that IL-1 drives the generation of pathogenic Th17 cells in experimental autoimmune diseases, including colitis (108–111). In contrast, Harrison et al. show that epithelial-derived IL-18 acts directly on CD4 T cells to limit colonic Th17 cell differentiation, partially through antagonizing IL-1R signaling (112). They also found that IL-18R1 signaling was critical for Foxp3 Treg cell-mediated control of colitis. These findings imply that IL-18 reduces intestinal inflammation by suppressing Th17 cells and promoting Treg function. Finally, in addition to producing active IL-1 and IL-18, inflammasome activation induces pyroptosis, the inflammatory form of programmed cell death, through gasdermin D. Currently it is unknown whether pyroptosis is involved in tumorigenesis. Recently, Wang et al. show that chemotherapy drugs induce pyroptosis through caspase-3 cleavage of gasdermin E (113). It would be interesting to know the role of pyroptosis and gasdermin D, and their relationship with IL-1 and IL-18 in tumor development and therapies.

### Inflammasomes Promote Cancer Development

#### Skin Cancer

Several reports indicate that inflammasomes and IL-1 promote inflammation-induced skin cancer in a two-stage carcinogenesis-induced papilloma model. Drexler et al. found that IL-1R- and caspase-1-deficient mice were partially protected against skin cancer induced by 7,12-dimethylbenz(a)anthracene (DMBA)/12-O-tetradecanoylphorbol-13-acetate (TPA) treatments (114). Caspase-1 and IL-1R KO mice have a later onset and reduced incidence of tumors after DMBA/TPA treatments, compared with WT mice. In an independent study, Chow et al. also found that NLRP3 KO mice exhibited reduced skin papilloma lesions in the inflammation-induced skin cancer model (115). These data suggest that inflammasome-dependent IL-1β production contributes to the development of epithelial skin cancer. Surprisingly, Drexler et al. found that mice fully deficient for ASC displayed a similar incidence of skin papilloma as WT mice. To dissect the role of the ASC molecule, they generated conditional KO of ASC in myeloid cells and keratinocytes. Results show that mice specifically deficient for ASC in myeloid cells are protected from DMBA/TPA-induced skin cancer, suggesting a tumor-promoting role of inflammasomes and IL-1R signaling in myeloid cells. In contrast, mice specifically deficient for ASC in keratinocytes develop more tumors, compared with WT controls, suggesting that ASC suppresses tumor development in keratinocytes. Furthermore, expression of ASC protein is lost in human cutaneous squamous cell carcinoma (SCC). These results indicate that ASC has complicated roles in tumor development: ASC functions as a tumor suppressor in keratinocytes, but a tumor-promoter in myeloid cells (114). Interestingly, Okamoto et al. demonstrated that late stage human melanoma cell lines constitutively synthesize and secrete IL-1β through activated NLRP3 inflammasome, which requires no exogenous stimulation. In contrast, early stage melanoma cells need a stimulation of IL-1R to induce the production of IL-1β (116). Moreover, the production of IL-1β by melanoma cells could be inhibited by the caspase-1 inhibitor or IL-1R blockade. The authors suggest that the inflammasome/IL-1 autocrine loop contributes to the phenotype and progression of human melanoma.

A recent study unveils a very exciting finding about genetic mutations of the NLRP1 inflammasome in some skin cancers (31). There are two overlapping genetic skin disorders: multiple self-healing palmoplantar carcinoma (MSPC) and familial keratosis lichenoides chronica (FKLC), whose symptoms include numerous ulcerative, hyperkeratotic nodular growths on plantar and palmar skin. FKLC and MSPC skin lesions clinically resemble rapidly growing benign proliferative epithelial skin lesions known as keratoacanthomas, but histologically display characteristics of well-differentiated SCCs (31, 117). Furthermore, affected patients have increased susceptibility to malignant SCCs. Elegant research work from Reversade’s lab establishes a connection between genetic mutations of NLRP1 gene and increased susceptibility to skin cancer in human patients (31). To identify the genetic mutations in these two skin disorders, Zhong et al. performed whole-exome sequencing on genomic DNA isolated from affected patients, and found gain-of-mutations within the NLRP1 locus. Previous work showed that that NLRP1 is the most prominently expressed inflammasome in human skin. Functional studies of these mutations reveal that gain-of-function mutations in NLRP1 increase susceptibility to skin cancer, and a unique regulatory auto-inhibition mechanism in the NLRP1 inflammasome. In WT NLRP1, the Pyrin (PYD) and LRR domains interact with each other to inhibit its self-oligomerization. However, MSPC and FKLC associated mutations change the structure of these two domains, leading to constitutive NLRP1 self-oligomerization and inflammasome activation (31). As a result, keratinocytes from affected patients display spontaneous inflammasome activation and IL-1 secretion. Therefore, NLRP1 mutants cause skin hyperplasia *via* paracrine constitutive active inflammatory signaling. Together, these studies demonstrate that germline, gain-of-function NLRP1 mutations cause skin cancer and skin disorders. This is also the first study that provides genetic and functional evidence linking inflammasome mutations with cancer development.

#### Breast Cancer

Our recent study has demonstrated that inflammasome and IL-1β play a critical role in promoting tumor growth and metastasis in breast cancer (30). Our results show that tumor growth is associated with elevated levels of IL-1β in tumor microenvironments in mouse mammary tumor models and human breast cancer tissues. To evaluate the impact of inflammasome activities on tumor growth and metastasis, we utilized an orthotopic mammary gland tumor model in WT and inflammasome deficient mice. Compared to WT mice, primary tumor growth and lung metastasis in inflammasome-deficient mice were significantly reduced. Similarly, we found that IL-1R KO mice had reduced tumor growth after injection of breast cancer cells. Collectively, these results suggest that the inflammasome and IL-1 pathway promote tumor growth and metastases of breast cancer. Our data also show that inflammasome activation promotes the infiltration of myeloid cells such as tumor-associated macrophages (TAMs) and myeloid-derived suppressor cells. Furthermore, blocking IL-1R signaling with an IL-1R antagonist or anti-IL-1R antibody inhibits tumor growth and metastasis accompanied by decreased myeloid cell recruitment. We also found that targeting the inflammasome/IL-1 pathway leads to reduced tumor growth and metastasis in the xenograft mouse model of human breast cancer cells (30). Our results suggest that inflammasome activation and IL-1β production in TAMs provides an inflammatory microenvironment promoting breast cancer progression.

In another study, Kolb et al. show that obesity-induced NLRC4 inflammasome activation contributes to breast cancer progression (32). Obesity is one of the major risk factors for tumor development, including breast cancer. It causes metabolic and inflammatory changes that favor tumor growth and progression. Kolb et al. found that the obesity-associated tumor growth depended on caspase-1, as caspase-1 KO mice had significantly reduced tumor growth under experimental obesity conditions. In contrast, caspase-1 KO and WT mice displayed similar tumor growth under normal weight conditions. The authors further show that obesity is associated with NLRC4 inflammasome activation and IL-1β production in myeloid cells, which in turn induces VEGF and angiogenesis. Furthermore, tumor-bearing mice treated with metformin inhibit obesity-associated tumor growth (32). Currently, it is unclear how obesity is linked to NLRC4 activation in this tumor model. Nevertheless, this study establishes a causal link between obesity, inflammasome activation, and breast cancer progression.

### The Complicated Roles of Inflammasomes in Other Types of Cancer

NOD-like receptors have many family members in human or mouse genome. It is not surprising that different inflammasomes may have different or opposite roles in tumor development depending on their expression patterns and tumor types. In addition to the tumors we discussed above, studies on other types of tumors are starting to emerge. For example, Daley et al. show that NLRP3 signaling drives macrophage-induced adaptive immune suppression in pancreatic carcinoma (118). Pancreatic ductal adenocarcinoma (PDA) is associated with very high mortality rate with no effective therapy options. PDA is also characterized with inflammatory and immunosuppressive tumor microenvironments (119–121). Daley et al. found that NLRP3 deletion was protective against PDA (118). Their results show that NLRP3 promotes the expansion of immunosuppressive macrophages in PDA, which inhibit antitumor T cell response. Inflammasome activation and IL-1 signaling are also implicated in the development of asbestos-induced mesothelioma (122, 123). On the other hand, Wei et al. found that inflammasomes might suppress the development of human liver cancer (124). Emerging evidence indicates that inflammasomes play an important role in non-alcoholic fatty liver disease, a spectrum of metabolic disorders ranging from steatosis (NAFL) to steatohepatitis (NASH) to cirrhosis. Moreover, hepatic steatosis or cirrhosis is strongly associated with the development of hepatocellular carcinoma (125, 126). While many studies demonstrate that inflammasomes contribute to the pathogenesis of liver diseases, some reports show that inflammasome activities can inhibit liver steatosis (99, 127, 128). Moreover, the role of inflammasomes in HCC is not well studied. By analyzing the expression of NLRP3 components in HCC tissues and corresponding non-cancerous liver tissues, Wei et al. showed that the expression of inflammasome components was significantly decreased or completely lost in human HCC tissues, and that the deficiency in inflammasome expression is positively correlated with the advanced stages of HCC (124). This correlative analysis suggests that inflammasomes may suppress the development of human liver cancer. However, further clinical and experimental studies are needed to determine the role of inflammasomes and effector cytokines in liver cancer initiation and progression.

### The Inflammasome-Independent Role of AIMs and ASC in Cancer

Recent progress also indicates that there is an inflammasome-independent role of inflammasome components, particularly AIM2 and ASC, in tumor development. Man et al. showed that AIM2 suppressed colon cancer development by regulating intestinal stem cell proliferation (26). AIM2 was initially identified as a tumor suppressor in melanoma (129). Man et al. found that Aim2-deficient mice were hypersusceptible to colon cancer development, but inflammasome-associated cytokine levels were generally not affected. The results further demonstrate that deletion of AIM2 leads to the expansion of tumor-initiating stem cells through aberrant Wnt/β-catenin pathway (26). Similarly, Wilson et al. also found that there was an inflammasome-independent role of AIM2 in suppressing colon cancer development through DNA-PK and Akt signal pathways (130).

It has been shown that ASC is overexpressed or silenced in some tumors. Previous studies report aberrant methylation of ASC in breast, gastric, and prostate cancer (131, 132). There have been also studies showing that ASC inhibits tumorigenesis in primary melanoma cells by NF-κB suppression and p53/p21-related cell apoptosis (132–134). This may allude to an antitumor role behind ASC. Treating primary mouse keratinocytes or the human keratinocyte cell line with UVB-induced ASC-dependent phosphorylation of p53 and expression of p53 target genes (31). These results suggest that ASC may suppress tumor cells through induction of apoptosis or *via* p53 activation. It also suggests that ASC could be a novel target for anticancer therapy.

### Perspectives

Although inflammasomes are essential for host defense against pathogens and contribute to autoimmune disease, their roles in tumor progression are much more complicated. The inflammasome has been shown to be either tumor promoting or tumor suppressive in various cancers. Thus, inflammasomes function as a double-edged sword in tumorigenesis as well as the anticancer immunity. We hypothesize that the exact role of an inflammasome depends on multiple factors, including its expression patterns, effector molecules, tumor types, and stages of tumor development. Tumor microenvironments and gut microbiota may also influence the function of inflammasomes and host immunity, eventually tumor progression (Figure 1). To better understand the critical role that inflammasomes in cancer, and to develop novel therapeutics, a number of questions need to be addressed: (1) how inflammasomes, such as NLRP1, NLRP3, and NLRC4, are activated during tumor growth and progression; (2) the regulation of inflammasomes by other signaling molecules or pathways and the implication of such interactions in tumor development; (3) effects of inflammasome activation in different cell types on tumor progression; (4) effects of each inflammasome pathway on host antitumor immunity and immunotherapy; and (5) dysregulation or mutations of inflammasome components in human cancer.

Our results suggest that targeting the inflammasome/IL-1 pathway in tumor microenvironments may provide a novel approach for the treatment of certain cancer, such as breast cancer. On the other hand, the immunomodulatory of inflammasome activities, particularly IL-18, can be harnessed for immunotherapy against cancer. IL-18 has been shown to play an important role in the induction of IFNγ production, increasing NK cell activity and T cell proliferation (135–140). Notably, those immunoregulatory activities of IL-18 are overlapped with that of IL-12. In fact, IL-18 and IL-12 can cooperate to induce optimal IFNγ production, Th1 responses, and NK cell activation in response to pathogens (141–143). As such, systemic injection of IL-18 protein and over-expressing IL-18 have been reported to enhance T cell response and NK cell function in several tumor models such as B16 melanoma (135–137, 141, 142, 144, 145). IL-18 also has been tested in clinical trials to treat different human tumors (146). Therefore, IL-18 alone or in combination with other therapeutic drugs may hold a promising potential for cancer treatment. Furthermore, IL-18-activated NK cells or Th1 cells could be developed as immune cell therapies against cancer.

## Author Contributions

Conception, writing, and review: MK and BG.

## Conflict of Interest Statement

The authors declare that the research was conducted in the absence of any commercial or financial relationships that could be construed as a potential conflict of interest.
